# Bipolar Radiofrequency Catheter Ablation for Ventricular Arrhythmias

**DOI:** 10.31083/j.rcm2305179

**Published:** 2022-05-16

**Authors:** Kenzaburo Nakajima, David Zweiker, Michael Spartalis, Donah Zachariah, Luca Limite, Giovanni Peretto, Antonio Frontera, Paolo Della Bella

**Affiliations:** ^1^Department of Arrhythmology and Cardiac Electrophysiology, IRCCS San Raffaele Hospital, 20132 Milan, Italy

**Keywords:** bipolar, catheter ablation, intramural ventricular arrhythmias

## Abstract

A minority of premature ventricular contractions (PVC) and ventricular tachycardias (VT) have an intramural origin, which represents a challenge for conventional radiofrequency ablation. Bipolar ablation has the potential ability to create deeper and more transmural lesions and has been demonstrated to be optimal treatment in these cases. Bipolar ablation carries a relatively low risk of complications and is effective in eliminating or reducing the burden of ventricular arrhythmias. Despite its utility and efficacy, the clinical use of bipolar ablation is limited, and B-RF technology is still investigational and not widely available. This article reviews the technique of bipolar ablation and all its advantages when applied to specific scenarios.

## 1. Introduction

Radiofrequency (RF) catheter ablation is well indicated for the elimination of ventricular arrhythmias. Typically RF ablation is unipolar (U-RF). The substrate relevant to certain PVCs and the re-entry circuit of ventricular tachycardias (VTs), especially in non-ischemic cardiomyopathy (NICM), may sometimes include not just of the endocardial and epicardial layers but also the intramural myocardium. Not only endocardial but epicardial ablation is relevant strategy in some cases. For NICM, however, 45% of the VT circuits have been found to be predominately located in the intramural myocardium [1]. Even in the era of contact force and irrigated catheters, performing U-RF ablation, results in limited lesion depths (5–6 mm). The outcome for NICM VT ablation is poor, as the critical substrate can often be located deep in the interventricular septum (IVS), at a plane beyond the lesions created by conventional endocardial U-RF ablation. Bipolar radiofrequency ablation (B-RF) in view of its ability to create deeper lesions has been proposed to overcome these limitations.

In this review, based both on updated published data and our center’s experience, we aim to address the clinical and technical aspects of B-RF.

## 2. Animal Studies and Lesion Creation

U-RF at higher power settings and prolonged applications durations may produce deeper lesions, but with increased risks of complications such as cardiac tamponade or intramural hematoma due to steam pops increase [2]. B-RF ablation creates deeper and more effective lesions. Computational as well as animal models have demonstrated the superiority of B-RF over U-RF in wall thicknesses up to 15 mm. The duration of ablation is reduced and lesions are longer and deeper [3, 4]. While most studies have shown a thickness threshold of 15 mm with a contact force of 10 g and an ablation time of 60 sec, transmurality has been demonstrated in wall thicknesses of up to 25 mm, with the use of 20 g force and RF time of 120 sec [4]. The catheter tip orientation is also a determinant for lesion size and depth. These are greatest with the use of irrigated active and ground tip catheters oriented perpendicularly to the tissue [5]. When compared to high-power sequential or simultaneous U-RF, these biophysical features of B-RF may boost the efficiency of ablation of deeply placed arrhythmic substrates and also reduce the likelihood of collateral injury [6].

## 3. Indication for Bipolar Ablation

The indication for B-RF is complex and is often performed in redo cases, and current guidelines [7] recommend it for such cases, but do not state a clear indication for the VT specifically arising from deep myocardium. In particular, the indications for the use of B-RF for PVC/VTs at our institution are [8]: (1) Prior unsuccessful ablation with U-RF for VT originating from IVS; (2) Deep substrate, as defined by the identification of a mid-wall scar, most commonly involving the IVS [9], by either preprocedural imaging, prior ablation, or consistent VT morphology at 12-lead ECG [8]. The following intra-procedural conditions are also met: (1) IVS wall thickness ≥5 mm and (2) Linear distance between the catheter tips on either side of the septum is <15 mm [5].

## 4. How to Set Up Bipolar Ablation

**Fig. 1.**
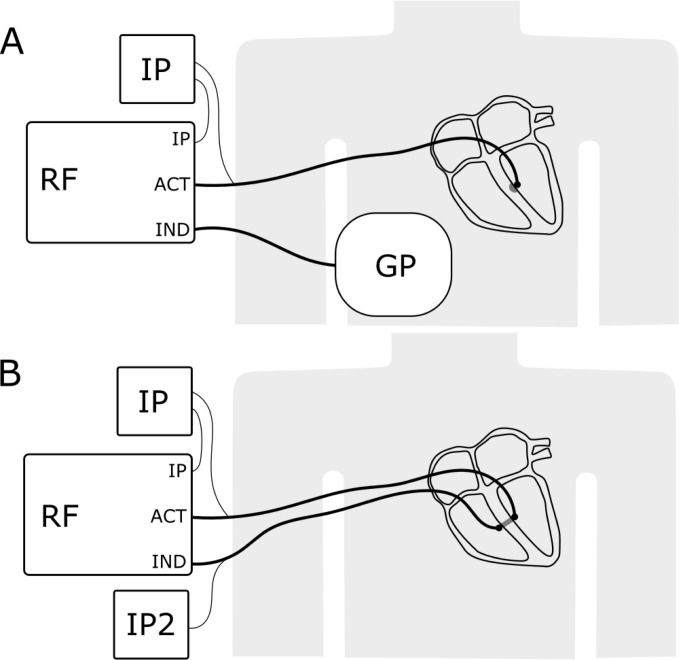
**Schematic setup of a usual unipolar irrigated radiofrequency ablation system (A) and a bipolar irrigated radiofrequency ablation system (B)**. In comparison to a unipolar ablation system, no ground pad is used, and a second ablation catheter is attached to the indifferent electrode port (IND) of the radiofrequency generator. ACT, Active electrode; IND, indifferent electrode; RF, radiofrequency generator; IP, irrigation pump; IP2, second irrigation pump; GP, ground pad.

Conventional U-RF delivers current between the catheter tip and a dispersive skin electrode. In B-RF, the dispersive skin electrode is replaced by a second ablation catheter that is close to the target area lying between the catheters (Fig. 1A). Thus B-RF can be applied not only at the IVS but also on either side of a papillary muscle [5], or between the endocardial and epicardial surfaces [7] or between coronary sinus or valve-endocardium in LV summit arrhythmia. It can be also utilised for the treatment of outflow tract [10], left ventricular (LV) summit [4, 11, 12, 13, 14] basal PVC/VTs [15], and even atrial arrhythmias originating from the mitral isthmus [16], cavo-tricuspid isthmus [4, 17] and inter-atrial septum [18].

### 4.1 Hardware

One open-irrigated catheter was connected to the standard port on the radiofrequency generator. To modify existing unipolar systems, a second ablation catheter is connected to the indifferent electrode port (Fig. 1B), instead of the cutaneous return patch, using a special cable that can either be custom-made or provided by the manufacturer for off-label use. A second irrigation pump (Fig. 1B) is then connected to the added catheter. Since the 2nd catheter is not connected to the RF generator, the 2nd irrigation pump needs to be operated manually at the same time as the ablation starts, which is one of the major limitations of current systems. Recently bipolar ablation adaptor from CoreSystem (Rzeszow, Poland) and RF generator irrigation pump from OSYPKA (Rheinfelden, Germany) that can control irrigation automatically and simultaneously during bipolar ablation are available in Europe.

### 4.2 Software

**Fig. 2.**
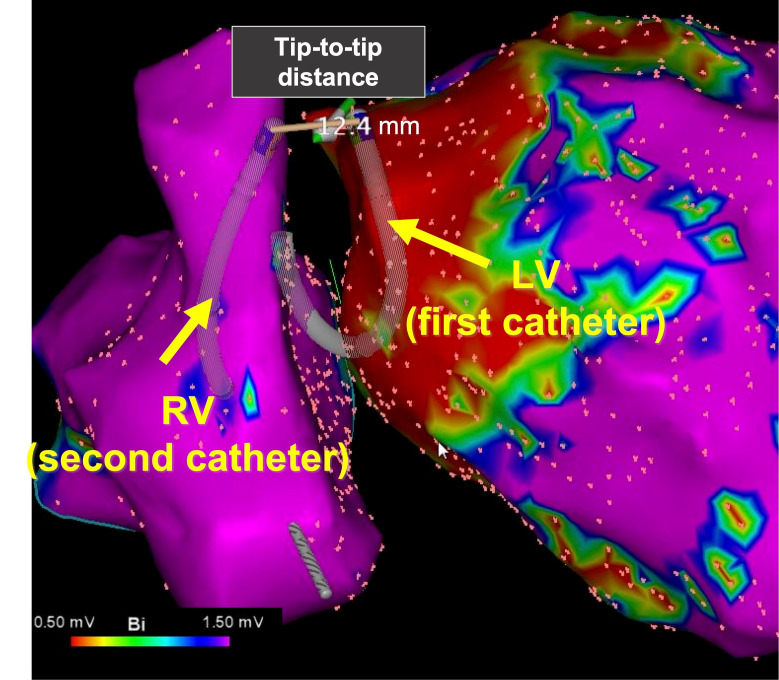
**Left anterior oblique view of bipolar ablation of a VT originating from the interventricular septum**. Both catheters are visualized as ablation catheters and the distance between them is displayed in mm (CARTO 3 system (Biosense Webster, Irvine, CA, USA)).

Only two 3D mapping systems (CARTO (Biosense Webster, Irvine, CA, USA), and EnSite (Abbott Medical, Abbott Park, IL, USA)) support bipolar ablation. Unipolar impedance cannot be measured in the primary or the secondary catheter for obvious reasons and would be irrelevant. While the second ablation catheter can be visualized on the CARTO mapping system, the typical parameters of an ablation catheter such as contact force, temperature measurements and ablation index are not available on the second catheter. With the EnSite mapping system (Abbott Medical, Abbott Park, IL, USA), the primary and secondary catheters can be displayed simultaneously on the EnSite Precision system but not on the EnSite X system and when visualized, the individual temperatures of the catheters and the impedance between the two tips can be recorded. Like U-RF, the use of B-RF should be supported with monitoring of indifferent electrode temperature to avoid adverse events related to possible overheating. In contrary, low temperature of indifferent electrode may suggest an insufficient effect of B-RF on the tissue in the contact [19]. A sample image of the bipolar ablation set-up from our centre, using the CARTO 3 system (which visualizes both ablation catheters) is displayed in Fig. 2. This case is the 50s-year-old man with non-ischemic cardiomyopathy who had been implanted with primary prevention CRTD. Total 4th sessions to ablate for VT were performed during this time. There were no abnormal electrograms in prior procedures and based on pace mapping the origin was found to be from the basal anterior of left ventricle (LV). Even after ablation he had received repetitive ICD shock therapies for VT, then we decided to perform catheter ablation with B-RF. VT was not induced under general anesthesia and it could not make an activation map of VT. We made a good pace-mapping from the basal anterior of LV and performed B-RF ablation without AV block (max tip to tip is 15.6 mm, max power 35W). Even after 1-year follow-up, there is no recurrence of VT.

To reduce the risk of complications and tissue damage, the position of the primary and the secondary ablation catheters should be planned beforehand and special caution taken when maneuvering the secondary catheter.

In clinical practice, the system is normally set up as a unipolar ablation system with the potential to switch to B-RF during the procedure. If U-RF is not sufficient, the secondary ablation catheter can easily be attached. Following this approach, all functionality from unipolar mapping and ablation (including unipolar impedance/voltage measurements) are available until the initiation of bipolar ablation.

It is important to be aware that, unlike U-RF, B-RF current is delivered uniformly via the two catheters and cannot be adjusted independently. This could be a disadvantage if a lower power is desired at either catheter site for safety reasons. Additionally, the effectiveness of B-RF can be compromised when the active and return catheters are in greatly different impedance environments (“impedance mismatch”), as the catheter operating in the higher impedance environment could constrain the amount of current that the system may deliver. The presence of epicardial fat or air at the end of one of the electrodes can also impact on the nature of lesions created as contact area is reduced and current density increased, resulting in lesions similar to those created by U-RF [20]. In our center, baseline impedance is in the range of 180–200 ohms, and we consider that impedance drops up to of 40 ohms are acceptable. However, bipolar impedance can be reduced by the use of 8 mm tip-electrode, and we may consider using the catheter with larger electrodes at times [21]. Furthermore, it is important to monitor the distance between the tips on a beat-to-beat basis.

### 4.3 Power Settings

There is no consensus about the optimal energy settings and duration of bipolar applications [6]. The choice of power settings should be guided by the nature of the substrate, such as the wall-thickness of the myocardium, histological heterogeneity of the targeted tissue [22], and anatomical considerations, such as proximity of the His-Purkinje conduction system and coronary vessels. Scar tissue is heterogenous and contains different amounts of cardiomyocytes, collagen fibers and adipose cells compared to normal healthy tissue. The effect of RF on scarred myocardium can be unpredictable. Therefore, it is useful to additionally evaluate physiological endpoints such as the impact of ablation on local electrograms, local non-capture confirmed by high output pacing and arrhythmic non-inducibility. Anatomical considerations as described above may warrant commencement of B-RF with low power settings with a view to increasing power provided there is no evidence of collateral damage. Typically, in our lab we use 35 W, 60–90 sec pulses.

## 5. Long Term Outcome of Bipolar Ablation

Several case series and case reports have demonstrated the feasibility of B-RF (Table 1, Ref. [4, 5, 8, 10, 14, 23, 24, 25]). As for the tip distance of the catheter, there was a case in which arrhythmia could be cured up to a maximum tip distance of 25 mm in thickness, and it was thought that a treatment strategy could be established as a guideline for the future [4]. The power output varied, but if the goal was to achieve IVS, the impedance drop was about 15–30 Ω for IVS at 30–50 W output. In this study, Futyma *et al*. [23] used 5% Dextro instead of saline as the perfusate and found no major complications. Most of the patients who underwent B-RF were complex or revision procedures having had previous unsuccessful U-RF ablation.

**Table 1.** **Clinical outcome of bipolar ablation for ventricular arrhythmias**.

Author (Year)	Site of origin of VT/PVC	N	Age	ICM	Distance between catheter tips (mm)	RF application Power	Mean RF application duration (seconds)	Impedance drop	Success rate (%)	Complications	Follow-up	Recurrence
			(years)	(N)		(W)		(ohm)		N	(Months)	N of pts (%)
Koruth *et al*., (2012) [4]	IVS	4	62	2	17.4 ± 3.3 (max 25 mm)	Mean 41 ± 7	198	32.5 ± 14.5	5 of 6 (83)	1 cAVB	Mean 12.8	2 (50)
	Free-wall	2	66	2					2 of 3 (66) (1 VT needed alcohol ablation)	0	12 and 17	0
Della Bella *et al*., (2020) [8]	IVS	21	66 ± 10	0	13 ± 3	Mean 33	60–90	27 ± 4	20 of 21 (95)	1 cardiac tamponade	Mean 25 ± 8	7 (33)
Futyma *et al*., (2020) [24]	Vicinity to His region	8	60 ± 15	0	-	Mean 35 ± 13 (10 to 60)	508 ± 565	-	6 off 8 (75)	0 (2 transient conduction block and 2 steam pops)	Mean 11 ± 5	VT 0
												PVC
												Pre: 16,200 ± 11,600 beats/day
												Post: 4500 ± 6200 beats/day (*p* = 0.035)
Nguyen *et al*., (2016) [5]	IVS	8	62 ± 6	7	-	30–40	-	16.9 ± 4.0	13 of 14 (93)	0 (1 cardiac tamponade, unrelated to procedure)	Mean 15 ± 6	2 (29)
	Papillary Muscle	2				Max: 50 (if both catheters are irrigation)		23.8 ± 8.0				1 (33)
						70 (if one is non-irrigation)						
Igarashi *et al*., (2020) [25]	IVS	11	65 ± 8	3	-	IVS: 30 to 45	30–1451	-	17/19 (89)	2 (1 (AVB, 1 Coronary stenosis))	12	5 (45)
	LV Free-wall	3				-				1 steam pop		2 (66)
	LVS	5				LVS: 20 to 40						1 (20)
Futyma *et al*., (2020) [23]	LVS	7	59 ± 12	0	-	36 ± 7	333 ± 107	NS 20–30	5/7 (71)	0	14 ± 6	VT 0
	(LPC-LVOT)					(2 cases used D5W)		D5W 50–70				PVC burden
												Pre 31 ± 13%
												Post 4 ± 5%
Futyma *et al*., (2020) [14]	LVS	4	55 ± 10	0	-	Mean 24 ± 6 (10 to 27)	244 ± 15	20–30	4/4 (100)	0	Mean 15 ± 4	VT 0
	(GCV/AIV-endo)				A safe distance (>5 mm) earliest site from coronary artery							PVC
												Pre 24,250 ± 1372 beats/day
												Post: 3000 ± 3600 beats/day (*p* = 0.02)
Teh *et al*., (2014) [10]	Outflow	4	53 ± 22	0	13.3 ± 5.4 (max 20 mm)	15–35	-	-	3/4 (75)	0	4	0


AFL, atrial flutter; AIV, anterior interventricular vein; cAVB, complete atrio-ventricular block; D5W, dextrose 5% in water; GCV, great cardiac vein; ICM, ischemic cardiomyopathy; IVS, Interventricular septum; LPC, Left pulmonary cusp; LVAD, left ventricular assist device; LVOT, Left ventricular outflow tract; LVS, left ventricular summit; N, number; NS, normal saline; PVC, premature ventricular contraction; VA, ventricular arrhythmia; VT, ventricular tachycardia.

With these the acute success rates with B-RF were very high. Ablation of PVCs reduced the burden on long-term follow-up, improved left ventricular ejection fraction and thus, prognosis. Nevertheless, the long-term recurrence rates of VT and complication rates are still suboptimal. For VTs with an interventricular septal origin, recurrence rates were 30–50% 1-year after using bipolar RF ablation powers of 30–45 W. One of the reasons for high recurrence rates was thought to be the depth of the intramural circuit arising from the deep substrate in the septum [4, 8]. Cardiac tamponade was recognized in one case and was successfully managed immediately. Complete AV block occurred in one case, but Futyma *et al*. [24] had excellent results for ventricular arrhythmias (VAs) in the vicinity of the His region (see detail in next paragraph). With VAs from an LV Summit origin, recurrence rates were quite low, even at long-term follow-up with no documented complications. In our center from 2016 to 2021, out of 26 patients septum (mean age 67 ± 10 years, 23/26 (88%) men; LVEF 37 ± 14%) with NICM and VT originating from the IVS who underwent B-RF, 7 patients (27%) had documented VT during 20 ± 11 months follow-up.

About intramural VTs, there are several alternative therapies such as ethanol injection and RF needle.

Although ethanol injection is limited by vascular anatomy, it can be used to treat deeper vessels. Coldwater may be injected before ethanol injection to check the effect. However, it is not possible to predict the degree of myocardial damage that will result from ethanol injection, and it is possible that other vessels may be injured [6, 26].

Needle ablation can be effective for deeper arrhythmic origins without anatomic limitations, but the risk of epicardial penetration and the fact that these techniques are not reversible may need to be considered [6, 26, 27]. The ablation efficacy of pulse-field ablation in the ventricle has been reported *in vivo*, and it is beginning to be reported that it can ablate deeper than conventional irrigation without damaging the AV block or other collateral sources [28, 29]. These reports indicate that the depth of the ablation layer increases with the number of applications, and the response to multiple applications to deep areas is promising.

## 6. Safety of Bipolar Ablation

The safety of B-RF remains a concern due to the potential for collateral damage. Clinical data available to date have reported the main complications to be conduction system disturbances (especially in septal ablation), cardiac tamponade, steam pop formation and coronary artery injury. These complication rates can be reduced if the following techniques are used:

(1) Careful titration of power and maintenance of the safest distance possible from sites of potential collateral damage. Futyma *et al*. [24] reported the safe ablation of PVCs in the vicinity of the His bundle by starting at 10 W, and titrating this as required (mean of 37 W with a range of 17–58 W in their case series), while ensuring a safe distance from the His bundle to the distal end of both catheter tips (evidence of a His signal at the proximal rings was accepted).

(2) Constant monitoring of impedance and distance between the two catheter tips is crucial. Steam pops and cardiac tamponade can be reduced by ensuring a perpendicular orientation for the catheter tips and cautiously titrating power according to impedance changes. Intracardiac echo in addition to the above measures would also be helpful [30].

(3) Coronary angiography where applicable, to ensure a safe distance from both catheter tips [25]. Futyma *et al*. [14] in their paper, described how for ablation at the LV summit, once a safe distance was confirmed between the coronary artery and the nearest site in the great cardiac vein, ablation was commenced with generator power settings in the great cardiac vein of 10 W, titrating up to 27 W, limiting catheter tip temperature to 39 °C with irrigation at 8–15 mL/min [14].

## 7. Future Advancement

For bipolar ablation, it is necessary to collect the number of cases in the future and set the appropriate output for each individual anatomy, especially in the vicinity of stimulation conduction. In addition, a new modality has recently been developed that does not interfere with the collateral source as easily as pulse field, but still provides sufficient therapeutic effect, and we look forward to the results of these studies.

## 8. Conclusions

B-RF is an optional therapy for VAs originating from deep substrates with an ability to create deeper lesions than U-RF safely and with excellent outcomes. However, it should never be forgotten that serious complications such as atrioventricular block or occlusion of vessels requiring PCI can occur because of deep ablation, which has a lower success rate for treatment.

Future research should address what the optimal power settings should be. Further investigations for safety and efficacy are key to acceptance as a conventional alternative ablation modality in specific scenarios as described previously.
